# Interconnecting District and Community Partners to Improve School-Level Social, Emotional, and Behavioral Health

**DOI:** 10.3390/bs15091225

**Published:** 2025-09-09

**Authors:** Kathryn B. Pohlman, Kayla Jones, Juan R. Lira, Jennifer Norton, Kelly Perales

**Affiliations:** 1 Midwest PBIS Network, Naperville, IL 60563, USA; juan.lira@midwestpbis.org (J.R.L.); jennifer.norton@midwestpbis.org (J.N.); kelly.perales@midwestpbis.org (K.P.); 2 West Kentucky Educational Cooperative, Murray, KY 42071, USA; kayla.jones@wkec.org

**Keywords:** coaching, social, emotional, behavioral, mental health, Positive Behavioral Interventions and Supports (PBIS), School Mental Health (SMH), Multi-Tiered Systems of Support (MTSS), Interconnected Systems Framework (ISF)

## Abstract

School districts face growing demands to address the academic, social, emotional, and behavioral health needs of all students, including meeting state mandates such as bullying prevention, suicide prevention, trauma response, and behavioral threat assessment. These needs have intensified since the COVID-19 pandemic, often resulting in fragmented and inefficient planning. The Interconnected Systems Framework (ISF) offers a structure for uniting district and community efforts into a single, integrated system of support. While research has expanded on the effectiveness of the ISF and resources have defined installation steps, the process is often arduous and challenging to notice progress and maintain momentum in action planning. This study examines the use of the ISF District–Community Leadership Team (DCLT) Installation Progress Monitoring Tool as a means to provide district and community leaders with concrete data to monitor progress and inform evaluation and action plans. Findings highlight the tool’s potential to strengthen installation processes, promote data-informed decision-making, and improve alignment of resources to impact student and school outcomes.

## 1. Introduction

The mental and behavioral health needs of children and youth have grown significantly over the past decade, with the COVID-19 pandemic intensifying existing challenges (Bommersbach et al., 2023; Centers for Disease Control and Prevention, 2024; Ghandour et al., 2019; Reinert et al., 2021). At the same time, schools face staffing shortages across both teaching and support roles, making it challenging to meet the increasing needs of students. Nationally, nearly half of public schools report unfilled vacancies, and the shortage of mental health professionals in schools and communities further limits access to critical supports (National Center for Education Statistics, 2022; Kaiser Family Foundation, 2024). Due to the increased need to support educators more intentionally, integrated systems, such as the Interconnected Systems Framework (ISF) are needed to ensure evidence-based practices are delivered with accuracy and accountability over time (Eber et al., 2019). Furthermore, tools such as the Installation Progress Monitoring Tool are necessary to align efforts between schools and communities, creating positive outcomes for both educators and students.

### 1.1. Understanding the Interconnected Systems Framework

In schools and other similar educational settings, two primary frameworks—Positive Behavioral Interventions and Supports (PBIS) and School Mental Health (SMH)—have shaped efforts to address students’ social-emotional-behavioral (SEB) needs. PBIS offers a tiered model of behavioral support, whereas SMH concentrates on providing a continuum of mental health services within schools. PBIS was introduced into policy with the reauthorization of the Individuals with Disabilities Education Act (1997) and established the four interactive elements of data, systems, practices, and outcomes as the base for implementation (Eber et al., 2019). Implementation of PBIS with fidelity has been linked to positive outcomes for students and educators. Studies show not only a reduction in exclusionary discipline, but also show improvement in engaged behavior and reading and math proficiency (Pas et al., 2019; Condliffe et al., 2022). SMH emerged in the 1990s due to the lack of mental health services, and provides locations within schools for students to receive mental health services (Eber et al., 2019). Mental health centers located within schools increase the likelihood that students with high-risk behaviors will seek out services (Bains & Diallo, 2015).

Although both have shown positive outcomes, they are often implemented in silos, resulting in fragmented services, duplicated efforts, and inefficiencies (Barrett et al., 2017; Weist et al., 2014). Schools implementing PBIS often do not reach full fidelity at advanced tiers (Sugai & Horner, 2020), School-based mental health services through co-located models are frequently isolated and lack coordination from school-wide efforts (Weist et al., 2022), and lack research to evaluate the effect on children and adolescents (Bains & Diallo, 2015).

The Interconnected Systems Framework (ISF) is an evidence-based, systems-change approach that unifies PBIS and SMH into a single, integrated delivery model by focusing on implementation science principles and PBIS as a Response to Intervention (RTI) (Barrett et al., 2017). Collaboration with both external experts and school district personnel is essential to developing and implementing new or reformed educational systems effectively (Skaar, 2022). Rather than operating separate systems for behavior and mental health, ISF supports a cohesive continuum of services by aligning leadership, teaming, data systems, and practices across school and community settings (Eber et al., 2019; Weist et al., 2022). The implementation science principles promote the consistent adoption of research findings and evidence-based practices into everyday use, to enhance the quality and effectiveness of the supports (Bauer & Kirchner, 2020). Key features include mental health providers becoming active members on school-based teams, universal screening and data-based decisions across all tiers, coordinated interventions with shared goals and language, professional development for both educators and clinicians, and a district-community leadership structure to guide the work (Eber et al., 2019).

Weist et al. (2022) found in a randomized controlled study that schools implementing the ISF, compared to those with only PBIS or co-located SMH services, delivered more Tier 2 and Tier 3 interventions and saw reductions in office discipline referrals and suspensions, particularly for students of color. ISF implementation was also associated with improved teaming structures, proactive student identification, and more effective planning involving both school and community partners (Weist et al., 2022). Overall expected outcomes of the ISF include improved social, emotional, behavioral, and academic functioning for students, increased access to mental health services, enhanced school climate and staff well-being, and reduced disproportionality in disciplinary practices (Eber et al., 2019).

### 1.2. Importance of the District Community Leadership Team in the ISF

Despite the increasing efforts to expand mental health support within school systems, sustaining these efforts beyond initial funding sources remains a significant challenge. In a systematic review of barriers and facilitators to sustaining mental health interventions, March et al. (2022) identified district infrastructure, coordination, and leadership as critical factors influencing the likelihood of continued school level implementation. Similarly, extensive research on the sustainability of other district-wide initiatives, such as PBIS, underscores the importance of administrator support as a strong predictor of sustained implementation (Coffey & Horner, 2012; McIntosh & Turri, 2014). Effective teaming has also been recognized as a key variable in predicting sustainability of PBIS (Mathews et al., 2014). Additional research emphasizes the importance of data-based decision-making and regular problem solving, high quality technical assistance, the capacity to adapt to school and community needs and culture, strong stakeholder relationships, effective training and coaching to implementers, family and student engagement, and creative funding solutions (Cook et al., 2019).

Building on these findings, Eber et al. (2019), identify the District Community Leadership Team (DCLT) as a critical component of successful ISF implementation. Ideally, the DCLT includes representation and authority from both district and community systems, with support from state agencies when available. This team lays the foundation for systemic transformation by using district and community data to guide decisions, align resources, and ensure that efforts are contextually relevant. A strong teaming structure and intentional collaboration are foundational to the successful installation and long-term sustainability of the ISF (Splett et al., 2017).

Swain-Bradway et al. (2015) define a DCLT as a cross-sector leadership group composed of school district leaders, representatives from youth-serving systems, and family groups. This group provides a foundation for district-wide implementation and scale-up. Drawing on the principles of implementation science, Eber et al. (2019) outline steps for district installation, led by the DCLT, which include using data to assess current systems, developing shared goals, and creating integrated action plans for training, coaching, and evaluation across both school and community systems.

The DCLT plays a pivotal role in bridging systems that have traditionally operated in silos, aligning efforts to improve student outcomes, and ensuring accountability and shared ownership across partners. Forming a DCLT is not just an initial step—the ISF defines it as a critical component for the sustained and effective implementation of ISF. Without a clearly defined leadership team, system change efforts risk becoming fragmented, inefficient, and ultimately ineffective (Eber et al., 2019).

### 1.3. The DCLT Installation Progress Monitoring Tool

Four stages of implementation of ISF are used to plan for sustainability and ongoing dialog and action planning. These four stages include exploration, installation, initial implementation, and full implementation (Eber et al., 2019). Although installation is a key stage of implementation with ISF, there is a scarcity of research, tools, and literature to determine the most effective way to install to maximize positive outcomes. While the other three stages are supported by data tracking tools such as the District Systems Fidelity Inventory (DSFI), Tiered Fidelity Inventory (TFI), and ISF Implementation Inventory, the installation stage has historically lacked such resources (Eber et al., 2019). Previous studies emphasize the importance of frameworks and implementation in phases, and barriers to implementation include the absence of tools (Leeman et al., 2018).

Coaches and leaders are supported in installing interconnected systems through five key steps: (1) forming a district/community executive leadership team; (2) evaluating the current status of mental health and PBIS systems; (3) building consensus around a shared mission statement; (4) establishing consistent team procedures and routines; and (5) developing action plans to guide and sustain implementation at demonstration sites (Eber et al., 2019). Although the steps appear in a linear order, the literature suggests that the actual work is not always linear, and some sites may complete later steps before the earlier ones (Eber et al., 2019).

The ISF DCLT Installation Guide is included as a resource in Volume 2: An Implementation Guide, which outlines the key steps, tasks, and activities required for a successful ISF installation. Designed as an action planning template, the guide serves as a roadmap for district-community coaches to support DCLTs in decision-making and establishing structures aligned with the ISF (Eber et al., 2019). The Midwest PBIS Network identified a gap in tools available to monitor the progress of district-level installation. While the ISF Implementation Inventory (Splett et al., 2019) supports school-level monitoring, there was a need for a complementary tool focused on district-level installation. In response, the ISF DCLT Installation Guide was expanded to serve as a planning resource and a progress monitoring tool for district-level ISF installation.

**Figure 1:**
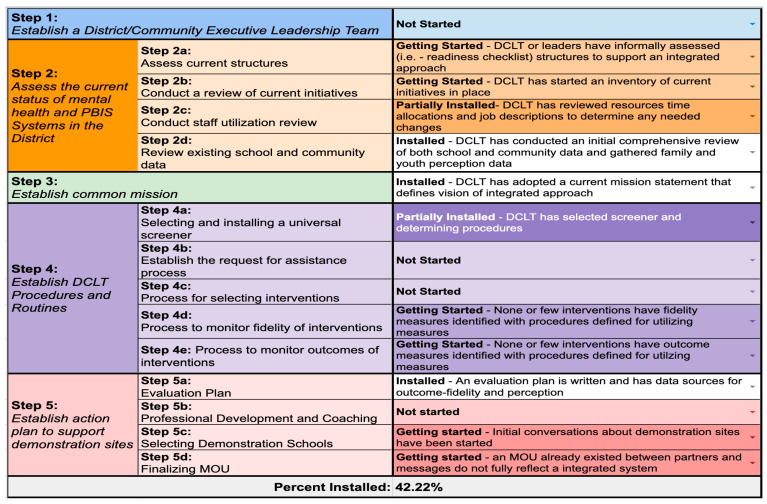
ISF DCLT Installation Progress Monitoring Tool—Version.

In the spring of 2022, the Midwest PBIS Network team of ISF technical assistance providers (TA providers) drafted the first version of the ISF DCLT Progress Monitoring Tool. Each step or sub-step on the ISF DCLT Installation Guide was defined into incremental installation phases (i.e., not started, getting started, partially in place, in place). See Table 1 for description of phases. The visual appearance for monitoring progress was also a critical point of discussion and design for the tool. The Midwest PBIS Network team wanted to view progress across multiple points of measurement quickly. To address this, the tool was formatted to include conditional color formatting, which darkens the shading based on progress. For example, “not started” was the lightest shade of the color based upon the step, and “in place” was the darkest shade of the color based upon the step. An example of the actual tool with visualization of installation progress is shown in Figure 1. The ISF DCLT Installation Progress Monitoring Tool addressed the gap in the research and resources to provide districts and schools with a data tracking tool to guide decisions and action planning, and determine readiness to advance into the next stage of ISF implementation.

## 2. Materials and Methods

### 2.1. Research Design

This study used a field-based, mixed-methods pilot design to examine the feasibility and utility of the ISF District–Community Leadership Team (DCLT) Installation Progress Monitoring Tool, which provided both data and a practical resource during the installation stage of ISF implementation. The pilot served as an initial step in evaluating the tool’s functionality in real-world districts, to inform tool refinement and readiness for broader implementation. The tool was tested across three projects involving school districts in Illinois, Kentucky, and New York. Three projects were included in the pilot study, which encompassed a total of 19 districts varying in size, demographics, and location. These 19 districts, spanning the three projects, represent a range of perspectives across the Midwest and eastern coasts of the US. Nearly half of all districts have household income below their state average (48% of districts) and/or the national average (57% of districts), with 62% of districts categorized as rural or town locale. All districts have a majority White student population, with two districts also serving nearly 25% of Black students in their area.

The first project involved twelve districts in Illinois participating in a two-year initiative (August 2022–June 2024) with Midwest PBIS Network aimed at expanding school-community partnerships using the ISF. Of these districts, 58% (7/12) are town or rural locales, with the remaining districts categorized as suburban locales. The number of schools in these districts varies from two to 12, with an average of five schools in each district. At least half of the districts have household income below both state and national averages.

The second project was a national model demonstration project (October 2021–September 2026), which provided training and coaching led by Midwest PBIS Network, focusing on strengthening school-family partnerships by installing an ISF in middle schools. Participating districts were from city, suburban, and rural locales with household income reflecting above, at average, and below versus state levels. This project provided the largest variation in the number of schools per district, ranging from three to 54, while also providing the most diverse student population, particularly in the Illinois school, where 24% of students are Asian, 15% are Hispanic or Latino, 4% are Black, and 2% are two or more races.

The third project involved the West Kentucky Educational Cooperative, a regional educational entity that provides support to 26 school districts. The cooperative was receiving support from the Midwest PBIS Network to establish processes that would support districts in installing ISF. Technical assistance providers from the cooperative began using the tool in August 2022with five districts, focusing on implementing PBIS through an integrated approach. The participating districts were located in rural Western Kentucky. They were categorized as city, suburban, town, and rural, with a range of five to 14 schools per district, averaging nine schools per district. All were public school districts with over 50% of students classified as economically disadvantaged, and a significant proportion eligible for free or reduced-price lunch. While all districts shared similarities in rural context and economic challenges, there was variation in ethnic composition; two districts had higher populations of Black and Hispanic students compared to others in the area.

The pilot was structured to explore both the feasibility and utility of the ISF DCLT Installation Progress Monitoring Tool. The team wanted to consider the extent to which the tool could be used effectively within routine implementation processes by TA providers. Secondly, the study aimed to assess the extent to which the tool and resulting data were perceived as helpful in monitoring progress, informing decision-making, and supporting implementation efforts.

### 2.2. Procedures for Collecting Data

Data collection took place over a three-year period (August 2022–May 2025), during which sites received regular technical assistance that included monthly coaching with district coach, coaching to DCLT, and support with evaluation activities, and the progress monitoring tool was completed at multiple time points. Projects One and Two both had established a schedule to update district progress three times per year, while Project Three was updated two times per year. Initially, in both Project One and Project Two, the tool was completed exclusively by Midwest PBIS Network TA providers, with no established process for sharing data with district coaches and/or teams. While the regional cooperative led the third project, the TA provider utilized the ISF DCLT Installation Progress Monitoring Tool alongside the district coaches to assess progress. Data was also aggregated from districts across projects to monitor variance in site progress.

Open-ended feedback from TA providers was gathered during project planning meetings. Comments were reviewed for recurring themes, and similar ideas were grouped to identify what made the tool practical, what adjustments needed to be made, and how teams used the data in reporting and planning.

## 3. Results

### 3.1. Feasibility Findings

The ISF DCLT Installation Progress Monitoring Tool was implemented across 19 districts during the first year of their project timelines. All 19 districts completed the tool at least twice in Year 1, with 15 (79%) completing it three times. In Year 2, one district exited the project. Among the remaining 18 districts, all completed the tool at least once: three completed it once, three completed it twice, and 12 completed it three times. In Year 3, among the seven districts with extended timelines, six completed the tool, with two completing it once and two completing it twice. A summary of tool completion frequency is presented in Table 2.

Across the 19 districts, 58% (11 districts) reached the 70% installation benchmark within two years. Among the four remaining districts with timelines extending beyond two years, 50% (two districts) met the benchmark within 2.5 years. These results include two districts with documented barriers related to district leadership support and one district that discontinued participation before reaching the two-year mark. At the two-year point, the median installation percentage was 73%, with a range from 0% to 91%, indicating wide variability in progress across districts. Project One, originally with twelve districts, had a schedule to complete the progress tool three times per year. It achieved 73% of districts (eight out of 11 districts, excluding the one district that discontinued the project after Year 1), reaching the 70% benchmark within two years.

Progress data showed substantial early gains. From baseline to the end of Year 1, districts saw an average installation increase of 36%, with project-specific ranges from 23% to 40%. From Year 1 to Year 2, the average installation gain decreased to 11%, with a range from 7% to 18%, reflecting a slower rate of change during sustained installation. Table 3 summarizes the average percentage increases in installation over time by project.

### 3.2. Utility Findings

An analysis of the Installation Progress Monitoring Tool was completed for each step after two years of monitoring. The following highlights were revealed through the analysis:

- Step 5c: Selecting Demonstration Sites—highest installation average (92%)
- Step 1: Establishing a DCLT—second highest average (90%)
- Step 2c: Conduct Staff Utilization—lowest average installation (28%)

Variability among each step also differed. The following highlights were revealed:

- The average range of difference between the highest and lowest installation scores per step was 24%
  ⚬ Step-specific variances ranged from 5.66% to 46.67%
- Step 2a: Assess Current Structures—least variability (6.66%)
- Step 2c: Conduct Staff Utilization Review and 4d: Process to Monitor Fidelity of Interventions—greatest variability (46.67% and 44.44%)
- Nine of the 11 districts (82%) reaching a 70% installation rate had both Step 1: Establish DCLT and Step 3: Establish Common Mission “In Place” at the two-year benchmark

Feedback from TA providers indicated that the tool was easy to use and supported meaningful reflection on implementation progress and action planning. During the first year of implementation, most providers reported using the tool independently, without direct involvement from district coaches or DCLT members. As implementation progressed, some providers began integrating tool completion into coaching sessions. Based on feedback from utilizing the tool with coaches, adjustments were made to turn the tool vertically, allowing for the visualization of progress to be displayed with DCLT and tracking of action steps. By the second year, many were incorporating both the installation percentages and visual displays from the tool into DCLT meeting discussions. Providers who used the tool collaboratively with coaches and teams noted that doing so appeared to accelerate team understanding and fluency with the installation steps, contributing to more engaged and informed implementation planning. “The progress monitoring tool served as our guide in keeping the district aligned and advancing toward our goals. The tool provided clear, actionable next steps that enabled collaborative planning with our district leadership team and provided a way to track and communicate progress with all stakeholders” (Henson, 2025).

### 3.3. Case Study: Graves County School District

Graves County School District, located in rural Western Kentucky, serves approximately 3600 students and employs over 250 certified educators. The district encompasses six elementary schools, one middle school, and one high school. In Spring 2022, the district initiated exploratory discussions with the West Kentucky Educational Cooperative (WKEC) to begin implementing PBIS through an integrated approach using the ISF.

To initiate the process, the district appointed a dedicated ISF District Coordinator and established a district implementation team. This team included key central office personnel such as two Instructional Supervisors, the Director of Pupil Personnel, and the Director of Special Education. A smaller leadership team met monthly to assess progress, plan next steps, and ensure effective communication across the broader team. The District Coordinator played a pivotal role in orchestrating implementation efforts through consistent communication and a commitment to sustaining the process.

In Fall 2022, the district began using the ISF District Installation Progress Monitoring Tool to assess baseline installation. With external coaching from WKEC, the implementation team reviewed this tool three times during Year 1, documenting progress, challenges, and next steps. By the end of Year 1, the district’s installation rate increased from 11% to 58%. As they saw their growth in installation progress, the transition to monitoring implementation was natural. At the end of Year 1, the team completed an initial baseline assessment of a district capacity tool for high-fidelity implementation of PBIS.

During Year 2, the team continued to monitor installation progress on a semesterly basis using the ISF District Installation Progress Monitoring Tool, along with ongoing technical assistance from WKEC, and increased their installation rate to 73%. The district capacity tool was also completed in the spring, demonstrating a 15% growth. By Fall 2024 (entering Year 3), the district had reached 80% on the ISF DCLT Progress Monitoring Tool and began to shift its focus toward enhancing implementation, guided by the district’s capacity tool. The district increased its implementation capacity score by 20% during Year 3.

These improvements reflect a significant investment in systemic change, sustained leadership, and alignment of social-emotional-behavioral initiatives across the district. The structured monitoring of the ISF installation served as a catalyst for strategic progress, supporting accelerated implementation.

## 4. Discussion

Findings from this field-based pilot study indicate that the ISF DCLT Installation Progress Monitoring Tool is a valuable support for guiding and assessing district-level installation of the ISF. When paired with technical assistance, the tool proved both feasible for use in real-world settings and useful in informing installation progress and planning. The tool fills a critical gap by providing resources to guide the Installation stage and inform next steps in ISF implementation, reinforcing the research behind the importance of both a framework and tools to successful implementation (Leeman et al., 2018).

High completion rates across all districts and project timelines suggest the tool is practical and beneficial to implement. All participating districts completed the tool at least once annually, with no significant decline in completion when used more frequently, up to two or three times per year. This consistent use, even in the absence of project mandates, suggests the tool is not overly burdensome and is perceived as helpful by both individuals and teams. Technical assistance providers’ feedback indicated that the tool enabled District Community Leadership Teams (DCLT) to create intentional action steps to support installation activities and decisions moving forward, aligning with multiple pieces of literature on successful DCLTs (Swain-Bradway et al., 2015; Eber et al., 2019).

Moreover, 58% of districts reached the 70% installation threshold within two years, underscoring the tool’s potential to track meaningful progress. In the case study district, completion of the ISF DCLT Installation Progress Monitoring Tool appeared to contribute to the team’s measurable growth in capacity assessment for monitoring implementation. This supports the literature in terms of using implementation science principles to enhance the quality and effectiveness of supports (Bauer & Kirchner, 2020). Feedback from TA providers further supports its utility in helping coaches and DCLTs communicate progress and plan next steps. The collaboration between schools, district leaders, and TA providers further reiterates the literature and the need for collaboration to implement reformed educational systems efficiently (Skaar, 2022). Based on these findings, it is recommended that districts using the ISF adopt a regular schedule—ideally two to three times per year—for completing the progress monitoring tool to support installation fidelity and momentum.

At a high level, completing a progress monitoring tool during the Installation stage of ISF implementation offers a systematic method for assessing readiness and guiding implementation decisions. The structured data generated helps teams identify strengths, address gaps, and prioritize next steps. By aligning actions with identified needs, the tool supports more strategic resource allocation and strengthens collaboration among district and community stakeholders, factors essential for sustainable ISF implementation.

### 4.1. Limitations

This study has several limitations. First, it did not assess the consistency and accuracy with which different users applied the tool’s criteria or interpreted the results. While TA providers were fluent in ISF district installation steps, a standardized schedule for tool administration and specific training on scoring and data use was not implemented across all sites. This may have affected inter-rater reliability and the consistency of how the tool informed action planning. Additionally, the absence of structured norming processes may have contributed to differences in interpretation and post-assessment use. The diversity of districts in this study were limited and only included districts from the United States. Finally, qualitative data from district participants was minimal. Future studies should incorporate training protocols and inter-rater reliability checks to enhance consistency in the application and scoring of the tool, include additional large, urban districts and international educational settings, and collect additional qualitative data from district participants.

### 4.2. Future Research

Although TA providers observed a relationship between strong district leadership and more rapid installation progress, this pilot was not designed to investigate that factor systematically. Notably, districts that reached the 70% installation benchmark within two years had commonly completed early foundational steps such as Step 1: Establishing a DCLT and Step 3: Establishing a Common Mission, both of which are indicators of leadership engagement. Future research should examine the direct impact of leadership commitment and team functioning on installation rates.

Additionally, the 70% benchmark used in this pilot was selected as a practical threshold for initial analysis but has not been empirically validated. Establishing a research-based benchmark for meaningful installation and studying its predictive validity for long-term implementation outcomes would be valuable. Continued follow-up with participating districts could also help determine whether early installation levels predict sustained implementation and improved system outcomes.

## 5. Conclusions

Findings from this field-based pilot study suggest that the ISF DCLT Installation Progress Monitoring Tool is both feasible to implement and useful for guiding the installation of key structures at the district-community level. High rates of tool completion across multiple time points, along with steady progress toward installation benchmarks, suggest that the tool can be effectively integrated into routine technical assistance and planning practices. TA providers found the tool easy to use and increasingly embedded it into coaching sessions and team meetings, supporting greater team fluency with the ISF. Variability in installation progress and step-level implementation suggests that targeted support is needed for specific areas and that the tool can help identify where that support should be directed. Overall, the tool shows promise as a practical mechanism for monitoring progress and strengthening district-level implementation of an integrated system of support for student mental health and wellness. By filling a critical gap in the literature, this tool offers districts and schools a practical, data-driven resource to guide action planning and decision-making, strengthening their capacity to implement and sustain effective practices within the ISF.

## Figures and Tables

**Table 1:** ISF DCLT Installation Progress Monitoring Tool—Incremental Installation Phases.

ISF DCLT Installation Progress Monitoring Tool—Incremental Installation Phases					
Step 1: Establish a District/Community Executive Leadership Team		Not Started	Getting started—A team of district and/or community leaders meets but without all stakeholders represented and no plan to expand	Partially Installed—Representative DCLT is established but not meeting regularly at this time	Installed—Representative DCLT meeting routinely with operating procedures
Step 2: Assess the current status of mental health and PBIS Systems in the District	Step 2a: Assess current structures	Not Started	Getting Started—DCLT or leaders have informally assessed (i.e., readiness checklist) structures to support an integrated approach	Partially Installed—DCLT has completed DSFI (or similar) and action plan that is more than a year old or not being used to guide work	Installed—DCLT has completed DSFI (or similar) and identified action steps
	Step 2b: Conduct a review of current initiatives	Not Started	Getting Started—DCLT has started an inventory of current initiatives in place	Partially Installed—DCLT has completed inventory of initiatives and identified areas for conversation and decisions	Installed—DCLT has identified action steps to organize-align and eliminate initiatives
	Step 2c: Conduct staff utilization	Not Started	Getting started—DCLT has started conversations about current status of roles within an integrated model	Partially Installed—DCLT has reviewed resources time allocations and job descriptions to determine any needed changes	Installed—Job descriptions have been modified to allow all school and community staff to work within an integrated system
	Step 2d: Review existing school and community data	Not Started	Getting Started—DCLT has started a review of both school and community data	Partially Installed—DCLT has conducted an initial comprehensive review of both school and community data	Installed—DCLT has conducted an initial comprehensive review of both school and community data and gathered family and youth perception data
Step 3: Establish common mission		Not Started	Getting started with conversations to understand current partners missions and goals	Partially Installed—DCLT has reviewed existing mission statement of all partners and prioritized need through consensus process	Installed—DCLT has adopted a current mission statement that defines vision of integrated approach
Step 4: Establish DCLT Procedures and Routines	Step 4a: Selecting and installing a universal screener	Not Started	Getting Started—DCLT is researching and determining fit of universal screener	Partially Installed—DCLT has selected screener and determining procedures	Installed—Schools have completed universal screening at least once
	Step 4b: Establish a request for assistance process	Not Started	Getting started—DCLT is examining current pathways to requesting support for both school and community systems	Partially Installed—Request for assistance process is in place for only one system (i.e., school or community partner)	Installed—A single request for assistance process is in place within one continuum of support
	Step 4c: Process for selecting interventions	Not Started	Getting started—DCLT has completed an inventory of interventions	Partially Installed—DCLT has made decisions about interventions to eliminate, modify, or add based upon gaps in intervention inventory and needs in school and community data	Installed—DCLT has consensus for a single continuum of interventions to prioritize for installing
	Step 4d: Process to monitor fidelity of interventions	Not Started	Getting started—None or few interventions have fidelity measures identified with procedures defined for utilizing measures	Partially Installed—Some (more than 50%) of interventions have fidelity measures identified with procedures defined for utilizing measures	Installed—All interventions have fidelity measures identified with procedures defined for utilizing measures
	Step 4e: Process to monitor outcomes of interventions	Not Started	Getting started—None or few interventions have outcome measures identified with procedures defined for utilizing measures	Partially Installed—Some (more than 50%) of interventions have outcome measures identified with procedures defined for utilizing measures	Installed—All interventions have outcome measures identified with procedures defined for utilizing measures
Step 5: Establish action plan to support demonstration sites	Step 5a: Evaluation Plan	Not Started	Getting started—Data sources are identified via a grant or another funding source but DCLT commitment and prioritization has not occurred	Partially Installed—Data sources are identified and prioritized without a formal plan or do not include data sources for outcome-fidelity and perception	Installed—An evaluation plan is written and has data sources for outcome-fidelity and perception
	Step 5b: Develop a plan for professional development and coaching	Not Started	Getting started—Initial conversations about needs and a PD and coaching plan have occurred	Partially Installed—Formal needs assessment and/or data analysis has occurred to begin prioritizing needs	Installed—A professional development and coaching plan is developed based upon an action and evaluation plan
	Step 5c: Selecting demonstration schools	Not Started	Getting started—Initial conversations about demonstration sites have been started	Partially Installed—Determining criteria for selecting demonstration sites is being defined	Installed—Demonstration sites have been selected
	Step 5d: Finalizing MOU	Not Started	Getting started—an MOU already existed between partners and messages do not fully reflect an integrated system	Partially Installed—MOU exists but key messages of integrated system are not fully reflected or MOU with some but not all partners	Installed—MOU reflecting integrated system messages is in place with all partners

**Table 2:** Frequency of Tool Completion Across Project Years.

Year	One Time	Two Times	Three Times	Total Districts
Year 1	0	4	15	19
Year 2	3	3	12	18
Year 3	2	2	0	7

**Table 3:** Average Increase in Items “Installed” on ISF DCLT Installation Progress Monitoring Tool.

Project	Baseline–Y1	Baseline–Y2	Baseline–Y3	Y1–Y2	Y1–Y3	Y2–Y3
Project One	↑ 40%	↑ 49%	NA	↑ 9%	NA	NA
Project Two	↑ 26%	↑ 32%	↑ 57%	↑ 7%	↑ 31%	↑ 25%
Project Three	↑ 23%	↑ 41%	↑ 51%	↑ 18%	↑ 28%	↑ 10%
Average	36%	41%	NA	11%	NA	NA

## Data Availability

The data presented in this study are available upon request from the corresponding author.
