# Correction: Pohlman et al. (2025). Interconnecting District and Community Partners to Improve School-Level Social, Emotional, and Behavioral Health. *Behavioral Sciences*, *15*(9), 1225

**DOI:** 10.3390/bs15101335

**Published:** 2025-09-29

**Authors:** Kathryn B. Pohlman, Kayla Jones, Juan R. Lira, Jennifer Norton, Kelly Perales

**Affiliations:** 1 Midwest PBIS Network, Naperville, IL 60563, USA; juan.lira@midwestpbis.org (J.R.L.); jennifer.norton@midwestpbis.org (J.N.); kelly.perales@midwestpbis.org (K.P.); 2 West Kentucky Educational Cooperative, Murray, KY 42071, USA; kayla.jones@wkec.org

In the original publication (Pohlman et al., 2025), the Data Availability Statement was missing. The Data Availability Statement has now been inserted as follows:

“Data Availability Statement: The data presented in this study are available upon request from the corresponding author.”

The authors state that the scientific conclusions are unaffected. This correction was approved by the Academic Editor. The original publication has also been updated.
